# Clinical characterization and mitochondrial DNA sequence variations in Leber hereditary optic neuropathy

**Published:** 2012-11-12

**Authors:** Manoj Kumar, Punit Kaur, Manoj Kumar, Rohit Saxena, Pradeep Sharma, Rima Dada

**Affiliations:** 1 Laboratory for Molecular Reproduction and Genetics, Department of Anatomy, All India Institute of Medical Sciences, Ansari Nagar, New Delhi, India; 2 Department of Biophysics, All India Institute of Medical Sciences, Ansari Nagar, New Delhi, India; 3 Dr. Rajendra Prasad Centre for Ophthalmic Sciences, All India institute of medical Sciences, Ansari Nagar, New Delhi, India

## Abstract

**Purpose:**

Leber hereditary optic neuropathy (LHON), a maternally inherited disorder, results from point mutations in mitochondrial DNA (mtDNA). MtDNA is highly polymorphic in nature with very high mutation rate, 10–17 fold higher as compared to nuclear genome. Identification of new mtDNA sequence variations is necessary to establish a clean link with human disease. Thus this study was aimed to assess or evaluate LHON patients for novel mtDNA sequence variations.

**Materials and Methods:**

Twenty LHON patients were selected from the neuro-ophthalmology clinic of the All India Institute of Medical Sciences, New Delhi, India. DNA was isolated from whole blood samples. The entire coding region of the mitochondrial genome was amplified by PCR in 20 patients and 20 controls. For structural analysis (molecular modeling and simulation) the MODELER 9.2 program in Discovery Studio (DS 2.0) was used.

**Results:**

MtDNA sequencing revealed a total of 47 nucleotide variations in the 20 LHON patients and 29 variations in 20 controls. Of 47 changes in patients 21.2% (10/47) were nonsynonymous and the remaining 78.72% (37/47) were synonymous. Five nonsynonymous changes, including primary LHON mutations (NADH dehydrogenase subunit 1 [*ND1*]:p.A52T, NADH dehydrogenase subunit 6 [*ND6*]:p.M64V, adenosine triphosphate [ATP] synthase subunit a (F-ATPase protein 6) [*ATPase6*]:p.M181T, NADH dehydrogenase subunit 4 [*ND4*]:p.R340H, and cytochrome B [*CYB*]:p.F181L), were found to be pathogenic. A greater number of changes were present in complex I (53.19%; 25/47), followed by complex III (19.14%; 9/47), then complex IV (19.14%; 9/47), then complex V (8.5%; 4/47). Nonsynonymous variations may impair respiratory chain and oxidative phosphorylation (OXPHOS) pathways, which results in low ATP production and elevated reactive oxygen species (ROS) levels. Oxidative stress is the underlying etiology in various diseases and also plays a crucial role in LHON.

**Conclusions:**

This study describes the role of mtDNA sequence variations in LHON patients. Primary LHON mutations of mtDNA are main variants leading to LHON, but mutations in other mitochondrial genes may also play an important role in pathogenesis of LHON as indicated in the present study. Certain alleles in certain haplogroups have protective or deleterious roles and hence there is a need to analyze a large number of cases for correlating phenotype and disease severity with mutation and mtDNA haplogroups.

## Introduction

Leber hereditary optic neuropathy (LHON) was first reported in a patient more than 150 years ago, but it was first described (OMIM 535000) as a distinctive clinical entity in 1871 by the German ophthalmologist Theodore Leber (1840–1917) [1,2]. The prevalence of LHON is estimated to be 1:50,000 and can occur at any age [3] with acute painless loss of central vision. LHON is bilateral in about 25% of cases and rarely unilateral [4-6]. If unilateral, the fellow eye is usually affected within 6–8 weeks. LHON is a maternally inherited disease and shows variable penetrance with a male preponderance of 86% [7]. LHON often progresses rapidly, which leads to severe visual loss with only little probability of visual recovery [8]. LHON is usually caused by mtDNA mutations residing in genes encoding subunits of complex I (component of mitochondrial respiratory chain). Ophthalmologic findings in LHON patients are variable, but classical LHON cases exhibit abnormalities like vascular tortuosity of the central retinal vessels, swelling of the retinal nerve fiber layer, a circumpapillary telangiectatic microangiopathy [9], and a cecocentral scotoma develops with variable preservation of peripheral vision. Early ophthalmologic changes can include hyperhemic optic discs, disc pseudoedema, and microangiopathy [10]. The disease finally leads to optic disc atrophy.

Nearly all patients worldwide carry one of three mtDNA pathogenic point mutations at positions NADH dehydrogenase subunit 4 (*ND4*):p.R340H, NADH dehydrogenase subunit 1 (*ND1*):p.A52T, and NADH dehydrogenase subunit 6 (*ND6*):p.M64V [8,11]. Other pathogenic mtDNA LHON variants have also been described in various studies, with some still awaiting full confirmation for pathogenicity [9,12], but mitochondrial NADH dehydrogenase subunit 1 (*MTND1*) and mitochondrial NADH dehydrogenase subunit 6 (*MTND6*) genes of mitochondria are thought to be “mutational hotspots” for LHON-causing mutations, in addition to primary LHON mutations [9,13,14]. Though there are several cases with primary LHON mutations, but in mitochondrial diseases the phenotype depends on various factors, such as threshold levels of wild-type mtDNA, tissue specific mosaicism, and mtDNA haplogroup. It has been shown that European mtDNA haplogroup J has genetic susceptibility to LHON [15], which suggests that certain alleles in certain haplogroups have deleterious or protective roles. Thus, each sequence variation needs to be studied or analyzed for the mtDNA background.

In the last two decades it has been shown that the primary mutations in complex I polypeptides lead to LHON, but this has not yet led to a satisfying explanation of the pathophysiological mechanisms; other mtDNA mutations may also lead to disease outbreak or modification of the phenotype in LHON patients. In this study we investigated patients presenting to ophthalmology clinic for subacute visual failure and suspected of having LHON. This study was planned with the aim to screen LHON cases for mtDNA sequence variations (PCR-DNA sequencing) and to assess how these variations can affect protein structure and function.

## Methods

### Clinical examination and selection of cases

Twenty clinically diagnosed LHON cases from northern India, presenting at Dr. Rajendra Prasad Centre for Ophthalmic Sciences (All India institute of medical Sciences, Ansari Nagar, New Delhi, India), were enrolled for this study after ethical approval from the institutional review board (IRB#IRB00006862). Diagnosis of LHON is mainly based on the exclusion of all other factors responsible for sudden vision loss. So all those factors were considered and ruled out before a diagnosis of LHON was made. Detailed family history of the patients and controls was taken, which included associated periocular pain, to differentiate from papillitis; use of tobacco or alcohol or chronic systemic medication was noted to rule out toxic optic neuropathy. Detailed systemic and neurologic examination was done to check the involvement of cranial and peripheral nerves. Patients with LHON typically present with acute or subacute, sudden, painless, central vision loss leading to central scotoma and dyschromatopsia. All patients underwent a complete ophthalmic examination, including visual acuity measurement, slit lamp observation of the anterior segment, indirect ophthalmoscopy, and applanation tonometry. All patients also underwent an MRI of brain and orbit and fluorescein angiography.

No patient reported any drastic changes in their diet or intake of any drug or exposure to any toxic agent or pollutant around the time of visual loss. All patients had normal erythrocyte sedimentation rate and syphilis serology. None of the patients reported myotonia, exercise intolerance, palpitations, cardiac conduction abnormalities, oral or genital ulcers, erythema nodosum, or somatic anomalies. Patients were followed up in a neuro-ophthalmology clinic. Clinical manifestation of LHON patients have been tabulated (Table 1). A total of 20 ethnically and age-matched normal individuals without any history of ocular disorders were enrolled as controls. These were blood donors at AIIMS who reported no symptomatic metabolic, genetic, or ocular disorders as found on an extensive questionnaire regarding family history, past medical problems, and current health. The control group for mtDNA sequencing consisted of 20 individuals (15 men, mean age 23.64±2.54 years and five females, mean age 20.78±3.65 years).

**Table 1:** Clinical phenotypes of LHON patients.

**Patient ID**	**Age of onset (in years)**	**Sex**	**Neuro- Imaging**	**VA** **OD OS**	**Fundus findings OD OS**	**Fields**	
						**OD**	**OS**
LHON 1	22	M	Normal	CF3ft CF5ft	diffuse disc pallor	Not possible	Not possible
LHON 2	25	M	Normal	20/80 20/50	diffuse disc pallor	central scotoma	central scotoma
LHON 3	27	M	Normal	CF5ft 20/60	diffuse disc pallor	Not possible	central scotoma
LHON 4	24	M	Normal	HMCF LP only	diffuse disc pallor	Not possible	Not possible
LHON 5	18	M	Normal	CF4ft CF5ft	diffuse disc pallor	Not possible	Not possible
LHON 6	26	M	Normal	20/200 20/100	diffuse disc pallor	central scotoma	central scotoma
LHON 7	28	M	Normal	CF5ft 20/60	temporal disc pallor	Not possible	central scotoma
LHON 8	30	M	Normal	20/80 20/50	diffuse disc pallor	central scotoma	central scotoma
LHON 9	23	M	Normal	20/200 20/100	diffuse disc pallor	central scotoma	central scotoma
LHON 10	24	M	Normal	CF1ft 20/100	diffuse disc pallor	Not possible	central scotoma
LHON 11	29	M	Normal	20/100 20/50	Severely diffuse disc pallor	central scotoma	central scotoma
LHON 12	25	M	Normal	20/80 20/50	diffuse disc pallor	central scotoma	central scotoma
LHON 13	22	F	Normal	20/200 20/100	diffuse disc pallor	Not possible	central scotoma
LHON 14	20	F	Normal	CF5ft 20/60	diffuse disc pallor	Not possible	central scotoma
LHON 15	28	M	Normal	20/200 20/100	diffuse disc pallor	central scotoma	central scotoma
LHON 16	24	M	Normal	20/100 20/50	Severely diffuse disc pallor	central scotoma	central scotoma
LHON 17	13	F	Normal	20/200 20/100	diffuse disc pallor	central scotoma	central scotoma
LHON 18	21	M	Normal	20/60 20/60	Severe diffuse disc pallor	central scotoma	central cecal scotoma
LHON 19	11	M	Normal	20/200 20/100	diffuse disc pallor	central scotoma	central scotoma
LHON 20	29	M	Normal	CF5ft 20/60	temporal disc pallor	Not possible	Not possible

Abbrevations: OD-right eye; OS-left eye; CF- Counting fingers; ft- distance in feet; HMCF- hand motions close to face; LP-Light perception; VA-Visual acuity.

### Sample collection and DNA isolation

Five milliliters of peripheral blood were collected in EDTA vacutainer tubes after obtaining written consent and stored at −80 °C until further use. DNA was extracted from whole blood samples of all LHON patients and controls using a standard phenol chloroform method.

### PCR amplification and sequence analysis of the mtDNA coding region

The entire coding region of the mtDNA was amplified in LHON patients and controls using 24 pairs of primers [16]. PCR amplifications for all primer sets were performed in a 30 μl volume containing 1.0 μl of 20 μM stock solution for each primer, 100 ng of genomic DNA, 1 unit of Taq polymerase (Banglore Genei, Karnataka, India), 0.1 mM of each dNTP, 4 μl of 10× PCR buffer (with 15 mM MgCl_2_), by means of 30 cycles of amplification, each consisting of 30 s denaturation at 94 °C, 30 s annealing at 56 °C, and 1 min extension at 72 °C. Finally, extension for 5 min at 72 °C was performed. Amplified PCR products were purified using a gel/PCR DNA fragment extraction kit (Geneaid Biotech Ltd., Sijhih City, Taiwan). Purified PCR products were sent for sequencing to Molecular Cloning Laboratories (South San Francisco, CA). The full mtDNA genome was sequenced except the D-loop as this is a hyper-variable region. All fragments were sequenced in both forward and reverse directions for confirmation of any nucleotide variation. All sequence variants from both LHON patients and controls were compared to the *Human Mitochondrial Reference Sequence* NC_012920 provided by the NCBI, using ClustalW2 (multiple sequence alignment program for DNA; European Molecular Biology Laboratory-European Bioinformatics Institute).

### Computational assessment of missense mutations

Two homology-based programs, Polymorphism Phenotyping (PolyPhen) and Sorting Intolerant From Tolerant (SIFT), were used to predict the functional impact of missense changes. PolyPhen structurally analyzes an amino acid polymorphism and predicts whether that amino acid change is likely to be deleterious to protein function [17,18]. PolyPhen scores of >2.0 suggest the polymorphism is probably damaging to protein function. Scores of 1.5–2.0 are possibly damaging, and scores of <1.5 are likely benign. SIFT is a sequence homology-based tool that sorts intolerant from tolerant amino acid substitutions and predicts whether an amino acid substitution in a protein will have a phenotypic effect [19]. SIFT is based on the premise that protein evolution is correlated with protein function. Positions with normalized probabilities less than 0.05 are predicted to be deleterious and those greater than or equal to 0.05 are predicted to be tolerated by SIFT.

### Comparative structure modeling

The knowledge of the three dimensional structure of the protein is essential to determine the implications of structural changes induced by mutations in the protein. When the precise structure for the protein is not available, the homologous protein with known structure (template) forms the essential prerequisite for developing the structural model for the protein employing comparative structure modeling approach. A BLAST search [20] was done to reveal the maximum sequence identity mitochondrial protein. A software package, MODELER 9.2 program [21] in Discovery Studio (DS 2.0; Accelrys Inc., San Diego, CA), was used for molecular modeling and simulation. The best model in terms of stereochemistry, torsional geometry, and energetics parameters was selected for further analysis. The selected model was refined by energy minimization followed by molecular dynamics (MD) simulations.

The model structures of mutants were generated by altering the corresponding residues in the model structure of wild-type protein, using the “Build Mutant” protocol in DS 2.0. The generated model of the mutant was refined by energy minimization followed by MD simulations similar to the wild-type model.

## Results

Eleven patients were positive for one of the pathogenic changes whereas no pathogenic change was present in controls. MtDNA sequencing revealed a total of 47 nucleotide variations in 20 LHON patients, out of which 21.27% (10/47) variations were non synonymous and 78.72% (37/47) nucleotide changes were synonymous (Table 2). Also 29 nucleotide changes were found in 20 controls, out of which 17.24% (5/29) were non-synonymous and 82.75% (24/29) were found to be synonymous (Table 3). In patients highest number of changes were present in complex I genes (54%; 25/47) followed by complex III (19.14%; 9/47), complex IV (19.14%; 9/47), and then complex V (8.5%; 4/47). Although the frequency of non-synonymous variations found in controls were approximately similar to the patients but the changes in controls were non pathogenic. All the nucleotide variations were homoplasmic. No primary LHON mutation or pathogenic mutations were present in controls. Age of onset of symptoms did not differ significantly in patients with and without pathogenic mutations in current study.

**Table 2:** Mitochondrial DNA sequence changes in LHON patients.

**S. No.**	**Nucleotide substitution**	**Codon change**	**Amino acid change**	**Locus**	**Type of mutation**	**Polyphen/SIFT score**	**Pathogenicity**	**Reported/ Novel**
1.	C3507G	ACC>ACG	p.T67T	ND1	SYN	NA	NA	mitomap
2.	C3741T	ACC>ACT	p.T145T	ND1	SYN	NA	NA	mitomap
3.	C3970T	CTA>TTA	p.L222L	ND1	SYN	NA	NA	rs28357973
4.	G4113A	CTG>CTA	p.L269L	ND1	SYN	NA	NA	mitomap
5.	*G3460A	GCC>ACC	p.A52T	ND1	NS	1.646/0.00	YES	mitomap
6.	T4703C	AAT>AAC	p.N78N	ND2	SYN	NA	NA	mitomap
7.	A4916G	CTA>CTG	p.L149L	ND2	SYN	NA	NA	mitomap
8.	A4944G	ATC>GTC	p.I159V	ND2	NS	0.468/0.29	No	mitomap
9.	T5004C	TTA>CTA	p.L179L	ND2	SYN	NA	NA	rs41419549
10.	C6290T	TAC>TAT	p.Y129Y	CO1	SYN	NA	NA	mitomap
11.	G6305A	GGG>GGA	p.G134G	CO1	SYN	NA	NA	mtDB
12.	T6320C	CCT>CCC	p.P139P	CO1	SYN	NA	NA	mtDB
13.	G6734A	ATG>ATA	p.M277M	CO1	SYN	NA	NA	rs41413745
14.	T6908C	TCT>TCC	p.S335S	CO1	SYN	NA	NA	mtDB
15.	A7843G	ATA>ATG	p.M86M	CO2	SYN	NA	NA	mitomap
16.	T7961C	TTA>CTA	p.L126L	CO2	SYN	NA	NA	mitomap
17.	G8701A	GCC>ACC	p.A59T	ATP6	NS	0.430/0.60	NO	rs2000975
18.	G8865A	GTG>GTA	p.V113V	ATP6	SYN	NA	NA	mitomap
19.	G9123A	CTG>CTA	p.L199L	ATP6	SYN	NA	NA	rs28358270
20.	T9068C	ATA>ACA	p.M181T	ATP6	NS	1.579/0.00	YES	mitomap
21.	C9540T	CTA>TTA	p.L112L	CO3	SYN	NA	NA	rs2248727
22.	G9966A	GTC>ATC	p.V254I	CO3	NS	0.293/0.46	NO	mitomap
23.	T10238C	ATT>ATC	p.I60I	ND3	SYN	NA	NA	rs28358275
24.	G10310A	CTG>CTA	p.T84T	ND3	SYN	NA	NA	rs41467651
25.	C10400T	ACC>ACT	p.T114T	ND3	SYN	NA	NA	rs28358278
26.	C10181T	TTC>TTT	p.F41F	ND3	SYN	NA	NA	mtDB
27.	G10589A	CTG>CTA	p.L40L	ND4L	SYN	NA	NA	rs2853487
28.	*G11778A	CGC>CAC	p.R340H	ND4	NS	2.608/0.00	YES	mitomap
29.	C12348T	CAC>CAT	p.H4H	ND5	SYN	NA	NA	Novel
30.	T12477C	AGT>AGC	p.S47S	ND5	SYN	NA	NA	rs28608480
31.	A12810G	TGA>TGG	p.W158W	ND5	SYN	NA	NA	rs28359174
32.	A12849T	GCA>GCT	p.A171A	ND5	SYN	NA	NA	Novel
33.	T12879C	GGT>GGC	p.G181G	ND5	SYN	NA	NA	mitomap
34.	C12906T	ATC>ATT	p.I190I	ND5	SYN	NA	NA	Novel
35.	T13020C	GGT>GGC	p.G228G	ND5	SYN	NA	NA	rs75577869
36.	T13151C	CTA>CCA	p.L272P	ND5	NS	0.175/0.21	NO	Novel
37.	T13281C	GTT>GTC	p.V315V	ND5	SYN	NA	NA	mtDB
38.	*T14484C	ATG>ACG	p.M64V	ND6	NS	2.504/0.01	YES	mitomap
39.	T14783C	TTA>CTA	p.L13L	CYB	SYN	NA	NA	mitomap
40.	C14950T	CAC>CAT	p.H68H	CYB	SYN	NA	NA	Novel
41.	G15043A	GGG>GGA	p.G99G	CYB	SYN	NA	NA	rs28357684
42.	A15061G	GGA>GGG	p.G105G	CYB	SYN	NA	NA	mitomap
43.	T15067C	TTT>TTC	p.F107F	CYB	SYN	NA	NA	mitomap
44.	T15097C	ATT>ATC	p.I117I	CYB	SYN	NA	NA	mtDB
45.	T15287C	TTT>CTT	p.F181L	CYB	NS	0.967/0.01	–	mitomap
46.	G15110A	GCA>ACA	p.A122T	CYB	NS	0.401/0.65	NO	rs28357685
47.	C15493T	CTC>CTT	p.L249L	CYB	SYN	NA	NA	mitomap

Abbreviations: *Primary LHON mutations, NA- Not applicable, SYN synonymous; NS-non-synonymous; ND1-NADH dehydrogenase subunit 1; ND2-NADH dehydrogenase subunit 2; ND3-NADH dehydrogenase subunit 3; ND4-NADH dehydrogenase subunit 4; ND5-NADH dehydrogenase subunit 5; CO1-cytochrome c oxidase I; CO2-cytochrome c oxidase II; ATPase6-ATP synthase subunit a (F-ATPase protein 6); ATPase8-ATP synthase protein 8; CYB-cytochrome B.

**Table 3:** MtDNA variations identified in controls. Polyphen and SIFT were used to predict the pathogenicity of non-synonymous changes.

**S. No.**	**Nucleotide substitution**	**Codon Change**	**Amino acid change**	**Locus**	**Type of Mutation**	**PolyPhen/SIFT score**	**Pathogenecity**	**Reported/ Novel**
1.	G3591A	CTG>CTA	p.T95T	ND1	SYN	NA	NA	mtDB
2.	C3780T	GGC>GGT	p.G158G	ND1	SYN	NA	NA	mitomap
3.	G3918A	GAG>GAA	p.E204E	ND1	SYN	NA	NA	mtDB
4.	A3933G	TCA>TCG	p.S209S	ND1	SYN	NA	NA	mitomap
5.	A4093G	ACC>GCC	p.T263A	ND1	NS	0.476/0.38	No	mtDB
6.	A4793G	ATA>ATG	p.M108M	ND2	SYN	NA	NA	mtDB
7.	A5351G	CTA>CTG	p.L294L	ND2	SYN	NA	NA	mtDB
8.	G6305A	GGG>GGA	p.G134G	CO1	SYN	NA	NA	mtDB
9.	G6962A	CTG>CTA	p.T353T	CO1	SYN	NA	NA	mtDB
10.	T7738C	ACT>ACC	p.T51T	CO2	SYN	NA	NA	mtDB
11.	G7762A	CAG>CAA	p.Q59Q	CO2	SYN	NA	NA	mtDB
12.	T8143C	GCT>GCC	p.A186A	CO2	SYN	NA	NA	mitomap
13.	G8251A	GGG>GGA	p.G222G	CO2	SYN	NA	NA	mtDB
14.	T8503G	AAT>AAG	p.N46K	ATP8	NS	0.090/1.00	No	mtDB
15.	G8584A	GCA>ACA	p.A20T	ATP6	NS	0.362/0.19	No	mtDB
16.	C8650T	CTA>TTA	p.L42L	ATP6	SYN	NA	NA	mtDB
17.	A8718G	AAA>AAG	p.K64K	ATP6	SYN	NA	NA	mtDB
18.	G8886A	AAG>AAA	p.K120K	ATP6	SYN	NA	NA	mitomap
19.	G10310A	CTG>CTA	p.T84T	ND3	SYN	NA	NA	mtDB
20.	T10873C	CCT>CCC	p.P48P	ND4	SYN	NA	NA	mtDB
21.	A11467G	TTA>TTG	p.L236L	ND4	SYN	NA	NA	mitomap
22.	G12372A	CTG>CTA	p.T12T	ND5	SYN	NA	NA	mtDB
23.	A12381G	CTA>CTG	p.L15L	ND5	SYN	NA	NA	mtDB
24.	G12406A	GTT>ATT	p.V24I	ND5	NS	0.299/0.72	No	mtDB
25.	C12498T	TTC>TTT	p.F54F	ND5	SYN	NA	NA	mitomap
26.	G12561A	CAG>CAA	p.Q75Q	ND5	SYN	NA	NA	mtDB
27.	G13204A	GTC>ATC	p.V290I	ND5	NS	0.710/1.00	No	mitomap
28.	G15172A	GGG>GGA	p.G142G	CYB	SYN	NA	NA	mtDB
29.	T15067C	TTT>TTC	p.F107F	CYB	SYN	NA	NA	mitomap

Abbreviations: SYN-synonymous; NS-non-synonymous; ND1-NADH dehydrogenase subunit 1; NA- Not applicable; ND2-NADH dehydrogenase subunit 2; ND3-NADH dehydrogenase subunit 3; ND4-NADH dehydrogenase subunit 4; ND5-NADH dehydrogenase subunit 5; CO1-cytochrome c oxidase I; CO2-cytochrome c oxidase II; ATPase6-ATP synthase subunit a (F-ATPase protein 6); ATPase8-ATP synthase protein 8; CYB-cytochrome B.

### SIFT and PolyPhen analysis

SIFT and PolyPhen analysis of all nonsynonymous changes from cases and controls revealed five pathogenic changes, including primary LHON mutations (p.A52T in ND1 protein; p.M64V in ND6; p.M181T in adenosine triphosphate (ATP) synthase subunit a (F-ATPase protein 6) [ATPase6]; p.R340H in ND4, and p.F181L in cytochrome B (CYB) protein; Table 2 and Table 3). Eleven patients were positive for either of these pathologic mtDNA nucleotide changes, but none of control harbored any pathogenic nucleotide change (Table 3).

## Discussion

This study enrolled 20 LHON patients who experienced acute or subacute, bilateral, persistent optic neuropathies characterized by central visual loss that occurred simultaneously or sequentially within a period of 1 year. In this study we report 54% variations in complex I (NADH dehydrogenase [ND] group of genes) of the electron transport chain (ETC) as found in earlier studies, which is approximately 50%–90% of LHON cases in different ethnic populations [22,23]. Primary LHON mutations have been considered a hallmark of LHON patients [24,25]. *ND1*:p.A52T mutation was present in two patients, *ND4*:p.R340H was present in six patients, and *ND6*:p.M64V was present in three patients. None of the patients with primary LHON mutations reported a multigenerational history compatible with maternal inheritance. Two patients had pathogenic mtDNA sequence changes (*ATP6*: p.M181T; *CYB*: p.F181L) other than primary LHON mutations, while others had no nonsynonymous mtDNA changes. In this study we found a greater number of synonymous changes in patients as compared to controls. Synonymous changes are often assumed to have no effect on protein structure and function. Studies have suggested that codon bias may be a mechanism of regulating gene expression levels [26]. The high number of synonymous changes may decrease the overall rate of translation as the choice of codon or which codon is preferred depends on which codon is translated more rapidly, timely, and effortlessly. Thus, a large number of synonymous variations may not alter amino acids due to degeneracy of code; however, it may affect the efficiency of translation machinery and thus may decrease the rate of ATP production.

Patients with no obvious mtDNA abnormalities might have no mitochondrial disease or they might have mtDNA abnormalities isolated to the optic nerve (tissue-specific mosaicism), conceptually similar to mitochondrial myopathies [27]. The other reason for this might be that the levels of mutant mtDNA are so low that it is not confirmed using PCR. However, these patients may have elevated oxidative stress comparable to patients with primary LHON mutations. The penetrance of optic neuropathies is incomplete, and males are affected five times more than females. Thus, it is believed that although mtDNA is the primary risk factor, there are other secondary factors that attribute to visual loss. Seventy-five percent of 14484 LHON pedigrees belong to European haplogroup J, and penetrance of optic neuropathy is increased by haplogroup-associated polymorphism. There is also increased penetrance of 11778 when it is in haplogroup J, but penetrance in the case of 3460 is not influenced by the mtDNA haplogroup. Only about one-third of individuals harboring one of these three mutations eventually develop LHON, and the penetrance varies among different families [28,29]. Therefore, identification of other factors affecting LHON penetrance would be of value in elucidating the pathophysiology of retinal neuron loss, as well as in searching for clues that might relieve visual loss or prevent the onset of LHON.

Comparative structure modeling was done for two nonsysnonymous changes (*ATP6*: p.M181T; *CYB*: p.F181L) as the other nonsynonymous changes were found to be nonpathogenic on insilico analysis (SIFT and PolyPhen). Nonsynonymous nucleotide changes in *CYB* and *ATPase6* gene in LHON have been described previously [30]. Mutations in *ATPase6* have been reported in various diseases, like primary congenital glaucoma [31], primary open angle glaucoma, neuropathy–ataxia–retinitis pigmentosa, and mtDNA-associated Leigh syndrome patients [31,32].

**Figure 1:**
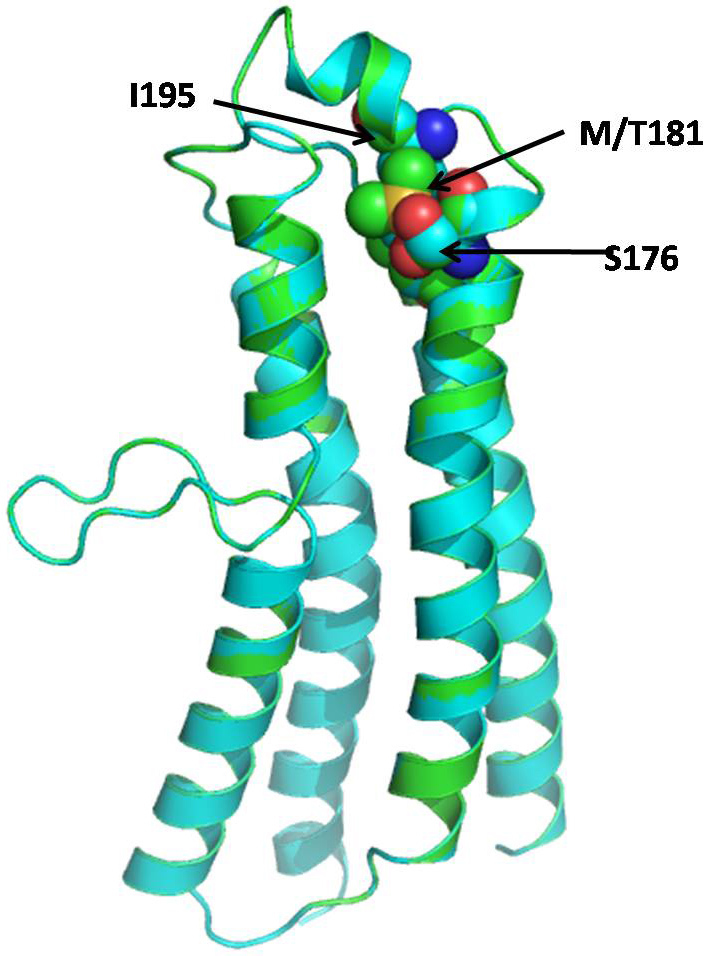
Superimposed structure of wild-type and mutant human mitochondrial ATP synthase subunit a (F-ATPase protein 6) (ATP6) in a ribbon. The side chain of Met181 is shown as a ball and stick.

**Figure 2:**
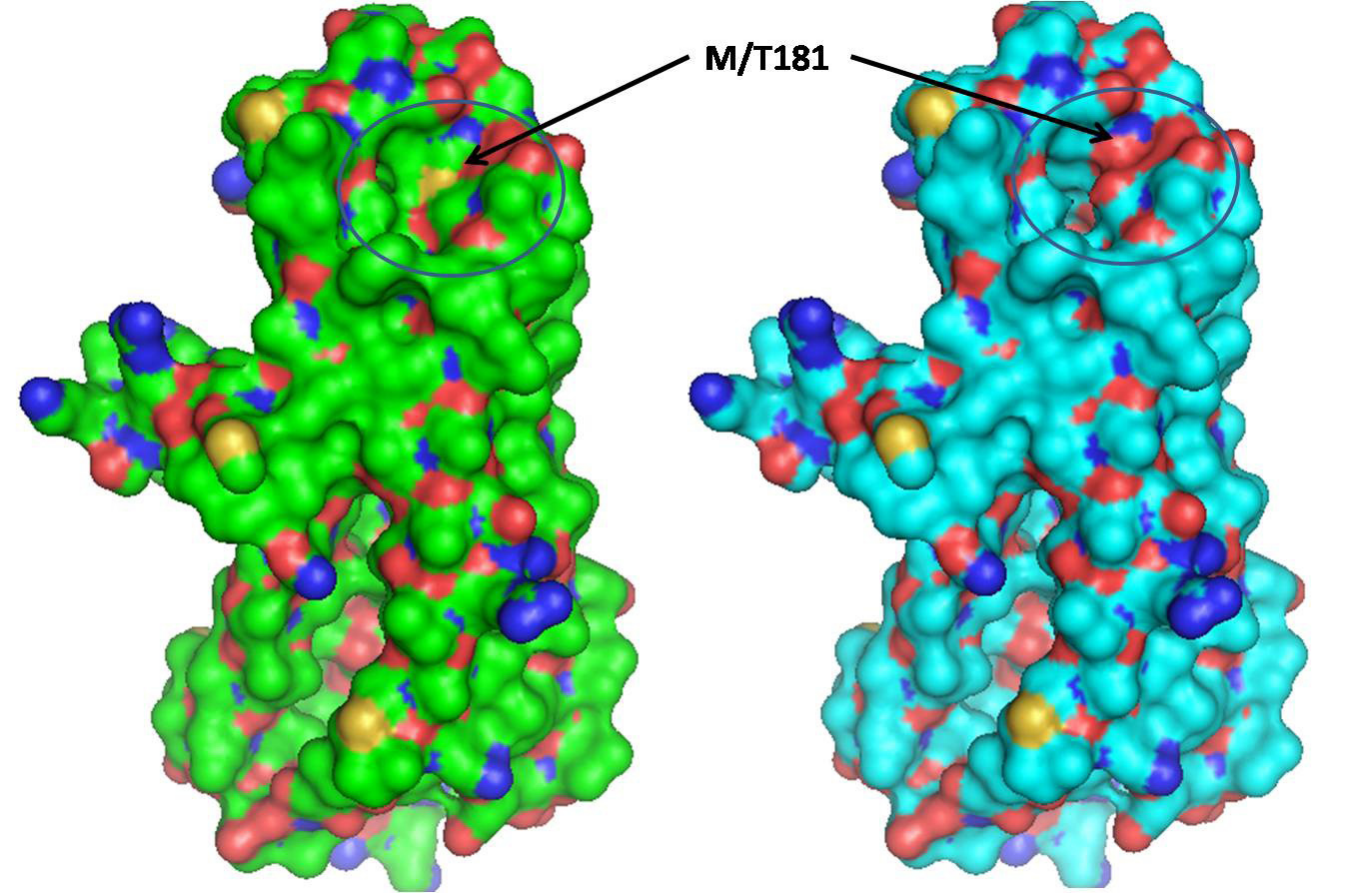
Surface structure of wild-type (green) and mutant (cyan) human mitochondrial ATP synthase subunit a (F-ATPase protein 6) (ATP6). Changes in M181T caused the changes in the surface cavity (shown by circles).

**Figure 3:**
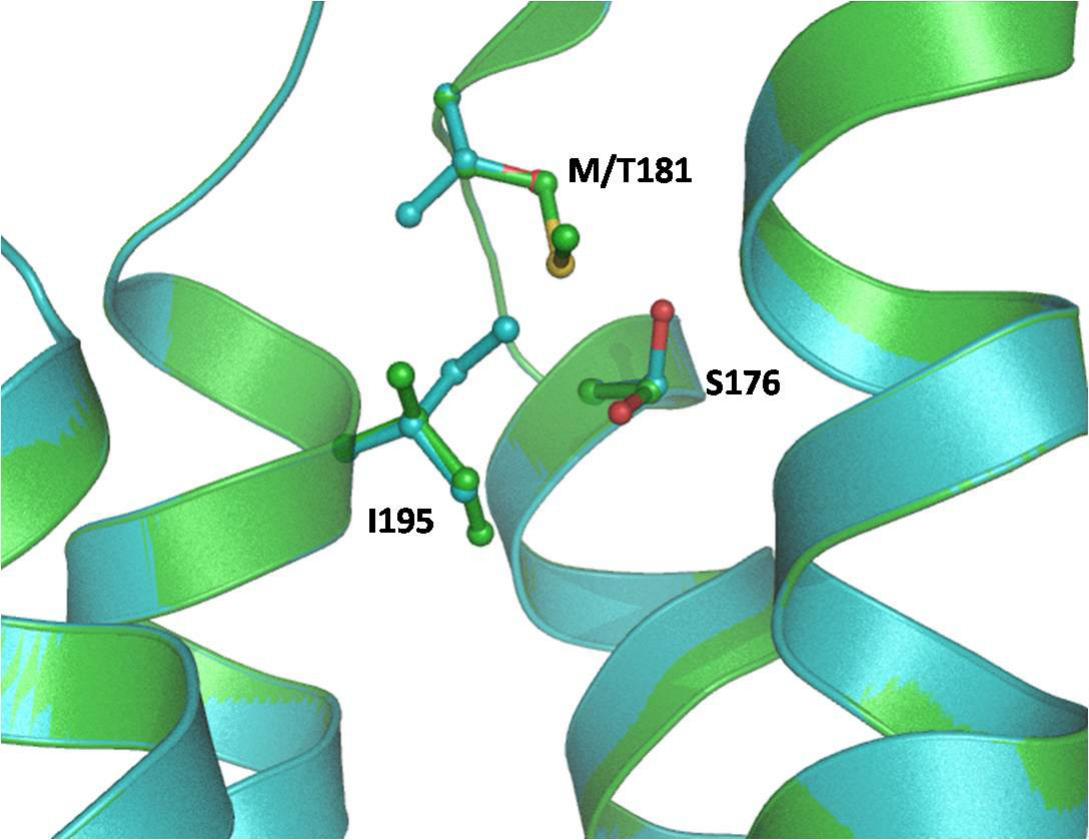
Model of wild-type human mitochondrial ATP synthase subunit a (F-ATPase protein 6) (ATP6) (green) superimposed on a model of M181T mutant (cyan). The side chain conformation of Met181, Ser176, and Ile95 (green) in the wild-type and Thr181, Ser176, and Ile95 (cyan) in the mutant are represented with the balls and sticks. The conformation of Thr181, Ser176, and Ile95 side chains were different in the native and mutant protein.

Mitochondrially encoded ATP synthase 6 is a subunit of the F_0_ complex of transmembrane F-type ATP synthase. ATP synthase comprises a rotary catalytic portion, F_1_-ATPase whose structure has been solved [33], a transmembrane portion F_0_, and two stalks that link F_1_ and F_0_. Two of the subunits of the F_0_ portion of ATP synthase, subunits 6 and 8 (or subunit a and A6L), are encoded in mtDNA in all animal cells [34]. This subunit (F_0_) is a key component of the proton channel and may play a direct role in the translocation of protons across the membrane [35]. The protein corresponds to ATP6 in the wild-type human mitochondria and consists of four helices and four connecting loops (Figure 1). The mutation Met181Thr is present in the helix of the protein, which is part of the binding pocket (Figure 2). The mutation replaces the Met residue, which is more hydrophobic compared to Thr and is also capable of making stacking interactions with neighboring residues. The Thr residue in the mutated protein is less hydrophobic than Met, the overall conformation of wild-type and mutant has changed due to the alteration of interactions with the neighboring amino acid residue Ser176 and Ile95 (Figure 2 and Figure 3). Since the Phe residue possesses a longer aromatic side chain, the Arg177 side chain shifts away from it to minimize steric hindrance and interacts with Ile164. The point mutation Met181Thr induces a conformational change in the Ser176 and Ile95 side chain orientation and positions it to interact with other neighbor residues (Figure 3). Since this mutation lies in the helix, it may affect its capability to interact with other subunit proteins of F_0_ assembly, which may lead to dysfunction of ATP synthase.

**Figure 4:**
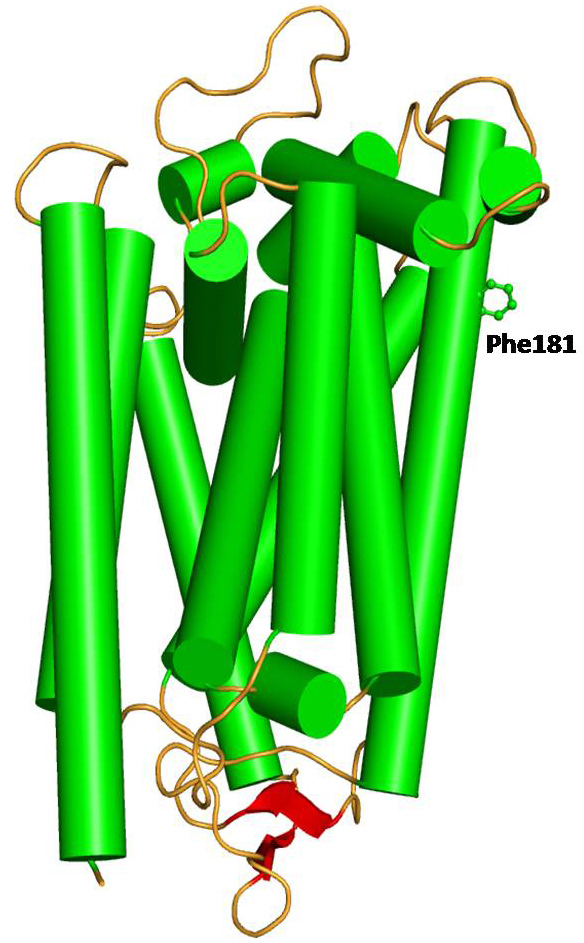
Model structure of wild-type human mitochondrial cytochrome b in cartoon rendering indicating secondary structure: helices (green), β-strand (red), and loop (orange). The side chain of Phe181 is shown as a ball and stick.

**Figure 5:**
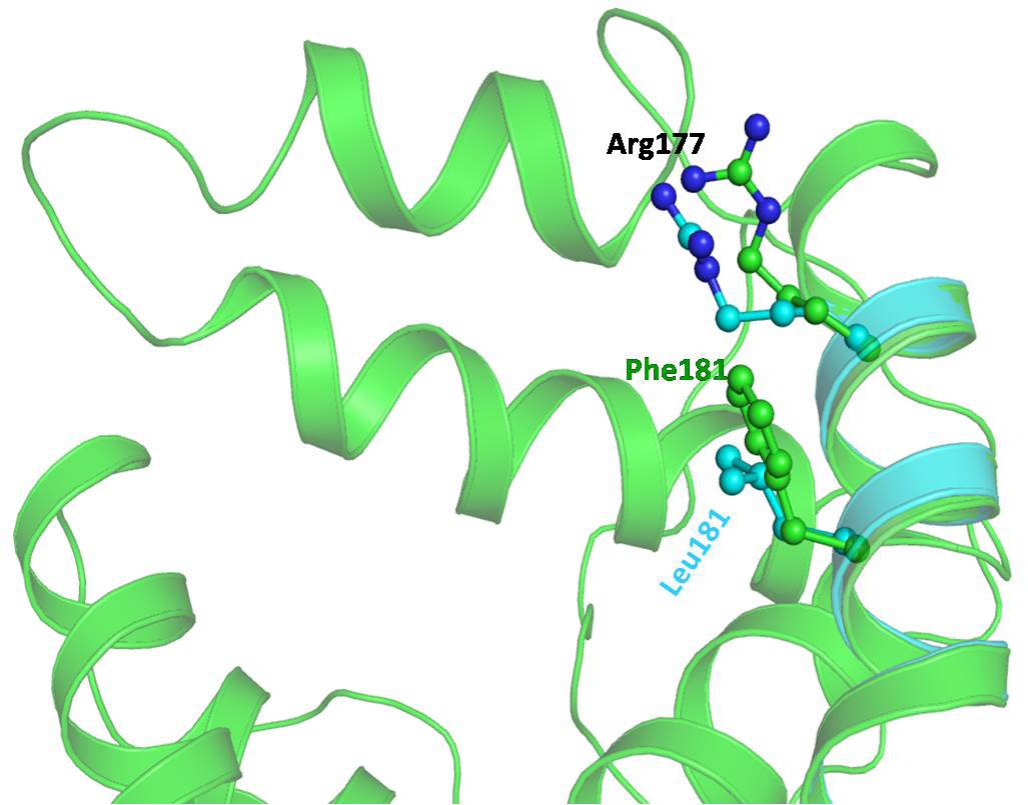
Model of wild-type human mitochondrial cytochrome b (green) superimposed on a model of F181L mutant (cyan). The side chain conformation of Phe181 and Arg177 (green) in the wild-type and Leu181 and Arg177 (cyan) in the mutant has been represented in the ball and stick. The conformation of the Arg177 side chain is different in the native and mutant protein. The Arg side chain in the wild-type adopts an orientation to minimize the repulsive force with Phe181.

Cytochrome bc1 (CYB) is a multisubunit membrane protein that has 11 subunits comprising three redox proteins: cytochrome b with two heme groups, cytochrome c1 possessing a covalently bound heme, and the iron-sulfur-containing protein with a [Fe_2_S_2_] cluster. The function of the other eight subunits in the mitochondrial protein is largely unclear. The CYB protein corresponds to cytochrome b in the wild-type human mitochondrial cytochrome bc1, which consists of 14 helices, two anti-parallel β-strands, and connecting loops (Figure 4). We found a mutation of Phe to Leu at position 181, which is present in the transmembrane region of the protein and is away from the binding pocket of the protein. The residue Phe possesses an aromatic hydrophobic side chain and is capable of making stacking interactions with neighboring residues. On the other hand Leu is hydrophobic in nature but shorter and lacks the capability of forming stacking interactions. Since both the residues are hydrophobic, the overall conformation of wild-type and mutant is conserved. A minor variation is, however, observed in the region of mutation (residues 174–185) due to the alteration of interactions with the neighboring amino acid residue Arg177. Since the Phe residue possess a longer aromatic side chain, the Arg177 side chain shifts away from it to minimize steric hindrance and interacts with Ile164. The point mutation to the shorter residue Leu induces a conformational change in the Arg177 side chain orientation and positions it to interact with residue Trp163 (Figure 5). This Phe181Leu mutation creates an empty space in this region leading to a decrease in hydrophobic interactions due to the shorter Leu and interruption of stacking capability in the mutant. Since this mutation lies in the transmembrane region, it will ultimately affect the capability of the CYB protein to interact with other interacting proteins.

Moreover, energy calculations of the wild-type and mutated proteins [36] (Poisson Boltzman with nonpolar surface area) indicates that the wild-type model has slightly lower energy (−11416 kcal/mol) compared to the mutant model (−11351 kcal/mol), signifying it is more stable than the mutant. This further substantiates the reduction of the capability of Leu181 to form contacts with neighboring nonpolar residues compared to Phe181 in the wild-type protein.

Many mechanisms have been studied and proposed as the bases for the pathogenesis of mitochondrial optic neuropathies. Abnormalities in mtDNA have been associated with LHON, primary open angle glaucoma (POAG), pseudo exfoliation glaucoma (PEG), primary angle closure glaucoma (PACG), primary congenital glaucoma (PCG), and other spontaneous optic neuropathies [16,31,37-39]. It is generally agreed that there are two main sites in the respiratory chain where superoxide anions are generated, which are complex I and complex III [40,41]. In the current study, complex I genes had 54% sequence changes. Neurons, because of their high energy requirement, are heavily dependent on mitochondria for survival [42]. Any malfunction of the mitochondrial electron transport chain results in excessive generation of free radicals and low ATP production. Oxidative stress (OS) has been suggested to play a crucial role in disease like glaucoma, LHON, proliferative vitreoretinopathies, and cataract [8,38]. Pathogenic mitochondrial mutations can cause mitochondrial dysfunction and enhance OS, which in turn leads to apoptosis in affected tissue and primary culture of human cells that harbor mtDNA mutations [43]. Oxidative stress-induced mtDNA damage has also been reported in other diseases, such as premature ovarian insufficiency [44,45], recurrent spontaneous abortions, and infertility [16,45].

Nonsynonymous mitochondrial variations adversely affect oxidative phosphorylation resulting in decreased mitochondrial respiration and increased free radical production [46]. This study highlights the role of nonsynonymous mutations and its effects on mitochondrial protein structure. Larger studies are required to report other primary or secondary mutations. The effect of a particular mutation in mitochondrial disease depends on its threshold level in particular tissue. The type of mutation and penetrance also vary among haplogroups as certain alleles in particular haplogroups have protective or deleterious effects. The etiology of LHON is complex, but the pathology is rather focal for a mitochondrial disease as a vast majority of patients have only optic neuropathy. Thus, we emphasize that the mutation spectrum should be analyzed in a large number of cases and in different haplogroups. Knowledge of mtDNA mutations and mitochondrial dysfunction in LHON may lead to a better understanding of optic atrophy in LHON. Novel approaches are now available for studying mitochondrial disease in the eye, and a novel in vitro treatment has already been devised for the metabolic defect of at least one mtDNA mutation in LHON [47]. It is crucial that further work and ideas are forthcoming to realistically treat or prevent the transmission of mtDNA disease to future generations. No generally accepted measures have been shown to either prevent or delay the onset of blindness in LHON. The long-term management of visually impaired patients remains supportive, with provision of visual aids and registration with the relevant social services.
